# Longitudinal association between muscle and bone loss: Results of US and Japanese cohort studies

**DOI:** 10.1002/jcsm.13438

**Published:** 2024-02-08

**Authors:** Yusuke Osawa, Yang An, Yukiko Nishita, Yasumoto Matsui, Marie Takemura, Eleanor M. Simonsick, Hiroshi Shimokata, Rei Otsuka, Hidenori Arai, Luigi Ferrucci

**Affiliations:** ^1^ Graduate School of Health Management Keio University Kanagawa Japan; ^2^ Longitudinal Studies Section, Translational Gerontology Branch, National Institute on Aging National Institutes of Health Baltimore MD USA; ^3^ Sports Medicine Research Center Keio University Kanagawa Japan; ^4^ Laboratory of Behavioral Neuroscience, Intramural Research Program, National Institute on Aging National Institutes of Health Baltimore MD USA; ^5^ Department of Epidemiology of Aging National Center for Geriatrics and Gerontology Aichi Japan; ^6^ Center for Frailty and Locomotive Syndrome National Center for Geriatrics and Gerontology Obu Japan; ^7^ Section of NILS‐LSA National Center for Geriatrics and Gerontology Obu Japan; ^8^ Graduate School of Nutritional Sciences Nagoya University of Arts and Sciences Nisshin Japan; ^9^ National Center for Geriatrics and Gerontology Obu Japan

**Keywords:** Aging, Cross‐country studies, Muscle strength, Osteoporosis, Sarcopenia

## Abstract

**Background:**

Muscle and bone are physiologically interconnected, but joint changes of muscle and bone with aging, and whether the muscle‐bone changes are different by sex and by country has been little studied. We examined longitudinal associations of bone mineral density (BMD) and muscle mass or muscle strength in community‐dwelling 65 years or older in the United States and Japan.

**Methods:**

The present analytic sample included 1129 women and men from the Baltimore Longitudinal Study of Aging (BLSA) (mean age, 74.5 ± 7.5 years; women, 49.8%) and 1998 women and men from the National Institute for Longevity Sciences‐Longitudinal Study of Aging (NILS‐LSA) (mean age, 70.0 ± 4.5 years; women, 51.4%). Median follow‐up was 4.6 (min‐max, 0–15.4) years in the BLSA and 4.0 (min‐max, 0–13.4) years in the NILS‐LSA. We selected visits at which participants had BMD (whole body, pelvic, femoral neck, trochanter, and Ward's triangle BMDs) and muscle mass [appendicular lean mass, (ALM)] measured by DXA scan. In each bone site, we ran cohort‐specific bivariate linear mixed‐effects models adjusted for baseline age, sex, body height, body weight, fat mass, education year, and smoking status. Race was an additional adjustment in the BLSA. Additionally, we performed sex‐specific analyses.

**Results:**

In the BLSA, the rate of change in ALM positively correlated with the rate of change in the whole body (rho = 0.30, *P* < 0.0001) and pelvic BMD (rho = 0.24, *P* < 0.0001), but not in trochanter, femoral neck, or Ward's triangle BMD (*P* > 0.05). In the NILS‐LSA, ALM positively correlated with the rate of change in all bone sites (rho ranged from 0.20 to 0.71, *P* < 0.01). In women, ALM positively correlated with the rate of change in all bone sites in both cohorts (in the NILS‐LSA, rho ranged from 0.35 to 0.91, *P* < 0.01; in the BLSA, rho ranged from 0.26 to 0.56, *P* < 0.05) except for femoral neck BMD in the BLSA. In men, ALM positively correlated with pelvic, trochanter, and Ward's triangle BMD in the NILS‐LSA (rho ranged from 0.45 to 0.68, *P* < 0.0001), and whole body and trochanter BMD in the BLSA (both, rho = 0.20, *P* < 0.05).

**Conclusions:**

Muscle loss co‐occurred with bone loss in both cohorts, but the association in the NILS‐LSA tended to be stronger than in the BLSA, and the association was higher in women than in men, implying that the association may differ by sex and country.

## Introduction

Physiologically, bone and skeletal muscle share many homeostatic signals, and accordingly, osteopenia and sarcopenia are considered interrelated musculoskeletal disorders. Indeed, these two pathological conditions share a genetic predisposition, sensitivity to mechanical stress, and endocrine system involvement.^1^ Osteopenia and osteoporosis are associated with a high risk of sarcopenia with the presence of these two conditions in the same individuals labelled osteosarcopenia.^1^, ^2^ The prevalence of osteosarcopenia increases with age and differs by sex, study setting (e.g., counties, community‐dwelling residents, and nursing home residents), and the operational definition of sarcopenia.^3^ Indeed, sarcopenia has been defined by different sets of criteria by different sarcopenia working groups and disagreement persists about the best definition.^4^, ^5^, ^6^ Depending on the diagnostic criteria for sarcopenia, individuals with the same physical functional status may or may not be diagnosed as having sarcopenia and its association with bone loss is not well understood. Elucidating the bone–muscle association in both men and women and different populations can examine the robustness of the association.

A cross‐sectional positive association between bone mineral density (BMD) and muscle mass has been found consistently across all age groups and appears to be stronger in men than women.^7^, ^8^, ^9^, ^10^, ^11^, ^12^ Only a few longitudinal studies have examined the association between bone mass and loss of muscle mass: in the Dubbo Osteoporosis Epidemiology Study (Australia), among community‐dwelling women and men aged 50 years and older, baseline lean mass was associated with differential rates of change in femoral and lumbar BMD during a 10‐year follow‐up period.^13^ In Chinese women aged 50–65 years, baseline muscle mass was positively associated with the rate of change in hip and lumbar spine BMD over a 2‐year follow‐up period.^14^ A very recent study using data from 1343 community‐dwelling Chinese men 40 years or older observed a positive longitudinal association of muscle mass with BMD over a 3‐year follow‐up period.^15^ Nevertheless, several knowledge gaps exist on the longitudinal relationship between BMD and skeletal muscle. First, no study has yet examined whether the rate of change in muscle mass is associated with the rate of change in BMD in either men or women. Second, no study has yet examined ethnic‐related differences in these associations. A study in the Health ABC cohort reported that the cross‐sectional association between muscle mass and BMD differed according to race and sex, which supports the need for further research examining longitudinal associations in different populations.^16^

We hypothesized that age‐related muscle mass changes co‐occur with age‐related change in bone, but the muscle–bone association over time might differ by country as well as other characteristics, such as sex, body size and composition, and bone region. To test this multifaceted hypothesis, we conducted a comprehensive analysis of longitudinal data from two cohorts of community‐dwelling older adults in the United States and Japan to explore potential divergences in the association between muscle mass and BMD across different populations and demographic factors. Because muscle strength is other component of sarcopenia, we also examined the longitudinal association between muscle strength and BMD. This project may help future interventions that may mitigate the impact of osteosarcopenia and enhance healthy‐longevity in older adults.

## Methods

### Participants

The Baltimore Longitudinal Study of Aging (BLSA) is a prospective open‐cohort study conducted by the National Institute on Aging, and the National Institutes of Health in the United States. The BLSA aims to describe normal aging processes in humans.^17^ BLSA participants undergo a 3‐day comprehensive examination, including body composition assessments and muscle strength tests. The interval between follow‐up visits depends on participants' age: 20‐ to 59‐year‐old participants are studied every 4 years, 60‐ to 79‐year‐olds every 2 years, and over 80 years every year. In the present analysis, we selected visits when participants were 65 years or older and had complete data on whole‐body BMD and appendicular lean mass (ALM, sum of arm and leg lean mass) assessed by a whole‐body dual‐energy X‐ray absorptiometry (DXA). Between September 2003 and September 2019, a total of 1129 BLSA participants (65‐ to 95‐year‐olds at first visit; women, *n* = 562; men, *n* = 567) were eligible for this study, providing 4256 person visits. The mean follow‐up time was 5.4 ± 4.4 years (median, 4.6 years; min‐max, 0–15.4 years). The BLSA protocol was approved by the NIH Intramural Research Program Institutional Review Board, and written informed consent was obtained from all participants.

The National Institute for Longevity Sciences‐Longitudinal Study of Aging (NILS‐LSA) is a prospective open‐cohort study conducted by the National Center for Geriatrics and Gerontology. The NILS‐LSA aims to describe the aging process and progression of diseases with aging.^18^ NILS‐LSA participants undergo a 1‐day comprehensive examination, including body composition assessments and muscle strength tests. The interval between follow‐up visits is every 2 years. In the present analysis, we selected visits when participants were 65 years or older and had complete data on whole body BMD and appendicular lean mass (ALM, sum of arm and leg lean mass) assessed using a DXA scan (QDR‐4500; Hologic, Bedford, MA, USA). Between November 1997 (first wave) and July 2012 (seventh wave), a total of 1998 NILS‐LSA participants (65‐ to 81 years old at first visit; women, *n* = 1027; men, *n* = 971) were eligible for this study, providing 6207 person‐visits. The mean follow‐up time was 4.5 ± 4.0 years (median, 4.0 years; min‐max, 0–13.4 years). Written informed consent was obtained from all the participants. The protocol of the NILS‐LSA was approved by the institutional ethics committee, and written informed consent was obtained from all participants.

**Figure 1:**
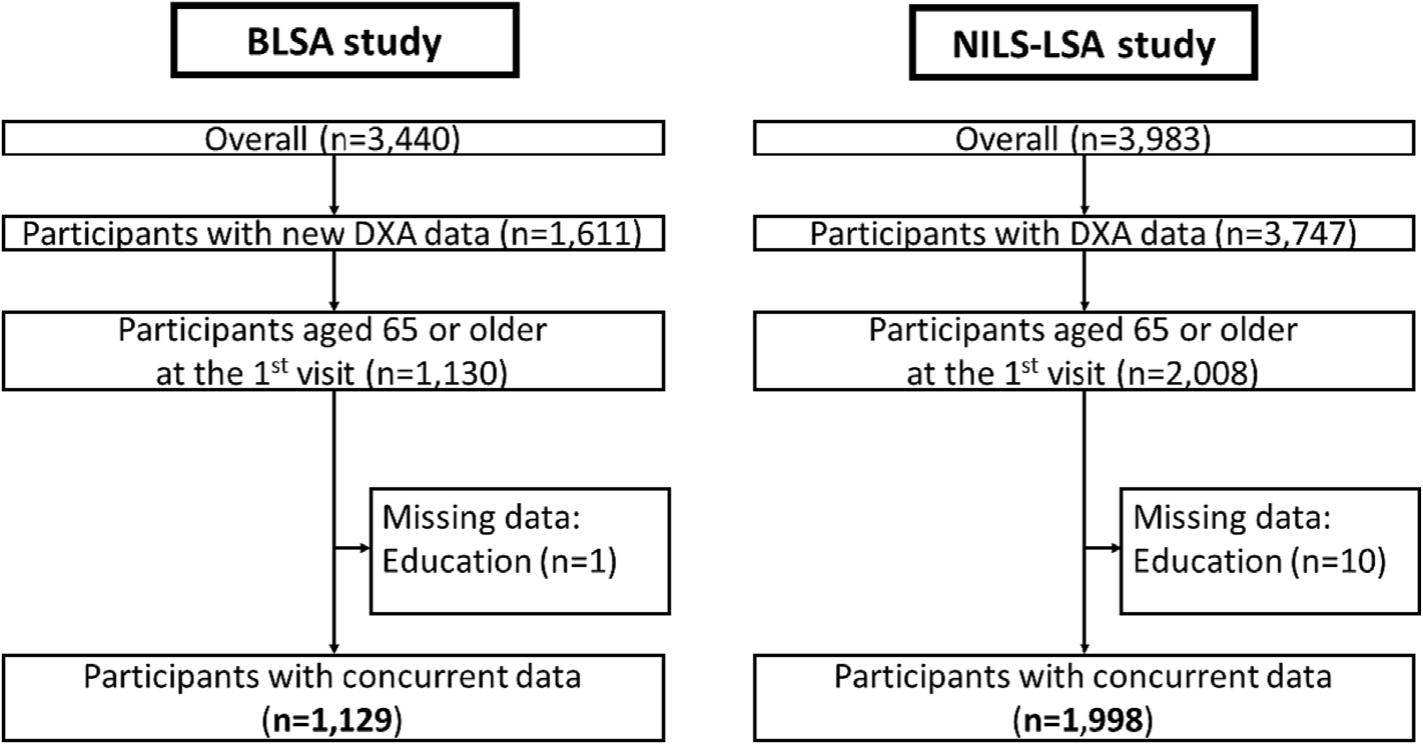
Flow of sample selection in the BLSA and the NILS‐LSA studies.

The flow of participant selection is shown in *Figure* *1*. The number of visits for each participant and histograms of follow‐up years in each cohort are shown in *Table* *S1* and *Figure* *S1*.

### Bone mineral density and body composition assessment

The BLSA measured whole body and regional BMD (spine, pelvis, rightward, right trochanter, and right femoral neck), ALM, whole‐body fat mass by DXA scan (Prodigy Dual Photon X‐ray Absorptiometry Unit, General Electric, Milwaukee, WI) with DICOM software ver. 10.51.006 with the array mode. NILS‐LSA study measured the same parameters by DXA scan with DICOM software V8.16a:3 for whole‐body and V8.20a:3 for regional bone sites.

### Isometric knee extension strength

The BLSA measured unilateral isometric knee extension peak torque using the Kin‐Com isokinetic dynamometer (Kin‐Com model 125E, version 3.2, Chattanooga Group, Chattanooga, TN, USA) from April 2003 until February 2011. From February 2010 to the present, the BLSA used the Biodex Multi‐Joint SystemPro dynamometer (Biodex Medical System, Advantage Software V.4X, Inc., Shirley, NY, USA). We used data collected with the two dynamometers after applying a conversion equation to harmonize the data.^19^ For both the Kin‐Com and the Biodex knee extension testing, participants were asked to extend their knee for 3 s as hard as possible for three trials with an instructor's verbal encouragement. The knee joint position was set at 120 degrees extension for the Kin‐Com and 70 degrees less than the full extension (130 degrees extension) for the Biodex. Participants rested for 15 s between trials. We used peak torque defined as the highest trial value for the right leg for the statistical analysis. The NILS‐LSA study measured unilateral isometric knee extension peak torque using a dynamometer (T.K.K.1281a, Takei Scientific Instruments, Niigata, Japan).^20^ The knee and hip joints were positioned at 90 degrees. The participants were asked to extend their knees for three trials, alternatively. The highest value of right leg was used for the analysis.

### Other covariates

Other covariates included sociodemographic variables [sex, age, race (the BLSA study only; black or not), and years of education], smoking status (current smoker or not), weight, and height. Weight was measured using a high‐precision mechanical scale. Standing height was measured to the nearest 0.1 cm. Given the likelihood of height loss due to age‐related disc reduction and change in posture, BMI may overestimate body size in older adults. Thus, we used height and weight as anthropometric covariates. We selected these covariates a priori because these covariates could confound the association between the outcomes.

### Statistical analysis

Descriptive data were reported as mean ± standard deviation (SD) or percentages. Cohort and sex differences were examined by independent *t*‐tests for age or chi‐square tests.

To examine cohort‐ and sex‐specific longitudinal change in ALM, BMD, and muscle strength, we performed linear mixed‐effects model, setting sex, baseline age, baseline age squared, education years, smoking status (current smoker or not), weight, height, whole body fat mass, time (follow‐up years), and race (black or not; the BLSA only) as fixed effects. The random effects included intercept and time.

Because of the heterogeneity of body size and composition between the two cohorts (*Table* *1*), we performed cohort‐specific bivariate linear mixed‐effects models to estimate the correlation between longitudinal changes in BMD and ALM.^21^ In these bivariate linear mixed‐effects models, longitudinal trajectories of BMD and ALM are modelled simultaneously. There are separate sets of fixed effects, random effects, and residual error terms for each outcome of the BMD and ALM parameters. For the BMD and ALM measures, in addition to the list of fixed effects described above, two‐way interactions between sex and baseline age with time were added as fixed effects and the same random effects as the linear mixed‐effects model above were included. We specified the 4 × 4 variance–covariance of the random effects to be unstructured. In the model, the correlations between the longitudinal change in BMD and ALM were directly estimated from this variance–covariance matrix setting as latent variables after accounting for fixed effects (rho ranges from −1 to 1). The bivariate linear mixed‐effects models were fit using PROC NLMIXED in SAS 9.4. We further examined sex‐specific analysis to estimate sex‐specific longitudinal associations in all analyses.

**Table 1:** Participants characteristics at the first visit.

	**BLSA study**			**NILS‐LSA study**		
	Overall	Women	Men	Overall	Women	Men
	(*n* = 1129)	(*n* = 562)	(*n* = 567)	(*n* = 1998)	(*n* = 1027)	(*n* = 971)
	Mean (SD),	Mean (SD),	Mean (SD),	Mean (SD),	Mean (SD),	Mean (SD),
	No. (%)	No. (%)	No. (%)	No. (%)	No. (%)	No. (%)
Age (years)	73.5 (7.5)	72.8 (7.6)	74.2 (7.3)	70 (4.5)	70.1 (4.5)	69.8 (4.5)
Sex (women, no. (%))	562 (49.8)	―	―	1027 (51.4)	―	―
Race (black, no. (%))	258 (22.9)	159 (28.3)	99 (17.5)	100% Asian		
Years of education (years)	17.6 (3.3)	17.3 (3.6)	17.8 (2.9)	11.1 (2.5)	10.8 (2.2)	11.5 (2.7)
Weight (kg)	76.5 (15.9)	69.6 (14.4)	83.3 (14.2)	55.6 (9.6)	51.2 (7.9)	60.3 (8.9)
Height (cm)	167.9 (9.2)	161.3 (5.9)	174.4 (6.8)	155.6 (8.8)	149.1 (5.6)	162.5 (5.8)
BMI (kg/m^2^)	27.0 (4.6)	26.7 (5.1)	27.3 (4.1)	22.9 (3.1)	23.1 (3.3)	22.8 (2.9)
Whole fat mass (kg)	26.7 (10.1)	28.2 (10.6)	25.3 (9.4)	15.5 (4.8)	17.1 (4.7)	13.7 (4.3)
Appendicular lean mass (kg)	20.6 (5.0)	16.6 (2.6)	24.5 (3.5)	16.2 (3.5)	13.5 (1.8)	19.0 (2.6)
Bone mineral density (g/cm^2^)						
Whole body	1.18 (0.13)	1.11 (0.11)	1.25 (0.12)	0.98 (0.13)	0.89 (0.09)	1.07 (0.10)
Pelvis	1.13 (0.16)	1.06 (0.13)	1.20 (0.16)	1.00 (0.15)	0.93 (0.12)	1.07 (0.15)
Femoral neck	0.89 (0.15)	0.85 (0.13)	0.93 (0.15)	0.66 (0.12)	0.61 (0.09)	0.72 (0.11)
Wards triangle	0.70 (0.16)	0.67 (0.14)	0.73 (0.16)	0.45 (0.13)	0.40 (0.12)	0.49 (0.13)
Trochanter	0.82 (0.16)	0.74 (0.13)	0.89 (0.16)	0.59 (0.12)	0.52 (0.09)	0.66 (0.12)
Follow‐up period (years)	5.4 (4.4)	5.4 (4.5)	5.4 (4.3)	4.5 (4.0)	4.3 (4.0)	4.7 (4.0)

We tested cohort or sex differences in each cohort in the association between the changes in BMD and ALM by the Wald test.

We also examined the longitudinal associations between BMD and muscle strength using the same sequence of analyses reported earlier. To better interpret the difference between the ALM–BMD association and the muscle strength–BMD association, we examined the longitudinal association between ALM and muscle strength as a supplemental analysis.

In addition, to explore the possible mechanisms of the bone–muscle association based on recent studies,^22^ we examined whether the plasma levels of IGF‐1 are associated with the rate of change in ALM or BMDs by using a sex‐specific linear mixed‐effect model in the BLSA.

Statistical Analysis Software (SAS) version 9.4 for Windows (SAS Institute, Inc., Cary, NC) was used for all data processing and statistical analyses, and R version 4.2.1. for forest plots. The level of statistical significance was set as *P*‐value < 0.05 (two‐sided).

## Results

Characteristics of the BLSA and the NILS‐LSA study populations are presented in *Table* *1*. Compared with the NILS‐LSA participants, the BLSA participants reported more years of education and were taller, heavier, and had higher muscle mass, fat mass, and BMD (*P* < 0.05). Additionally, BLSA participants were either white or black (22.9%), whereas NILS‐LSA participants were 100% Asian (Japanese).

### Longitudinal change in bone mineral density, appendicular lean mass, and muscle strength

With the exception of muscle strength in female participants in NILS‐LSA, BMD, ALM and muscle strength declined over time in both cohorts and sexes (*Table* *S2*).

### Longitudinal associations between bone mineral density and appendicular lean mass in overall samples

**Figure 2:**
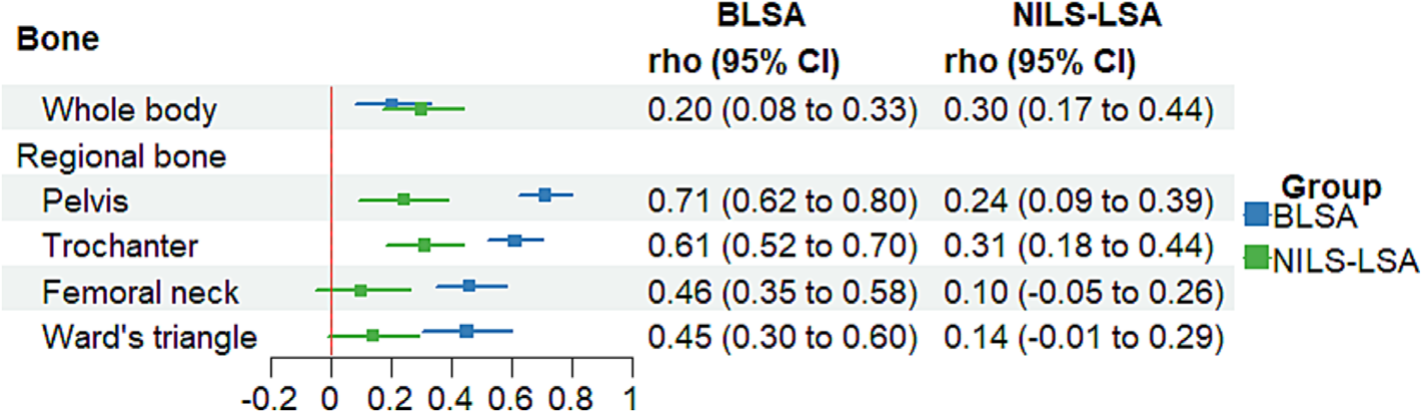
Results from cohort‐specific bivariate linear mixed‐effect model for the association between BMDs and appendicular lean mass in overall participants in the BLSA and NILS‐LSA studies. Models were adjusted for age centred at 70 years, age squared, sex, education year, weight, height, fat mass, smoking status, follow‐up time, sex*follow‐up time, and age*follow‐up time. In the BLSA study, models were further adjusted for race (black or not). Filled circles and bars indicate rho and 95% confidential interval.

**Figure 3:**
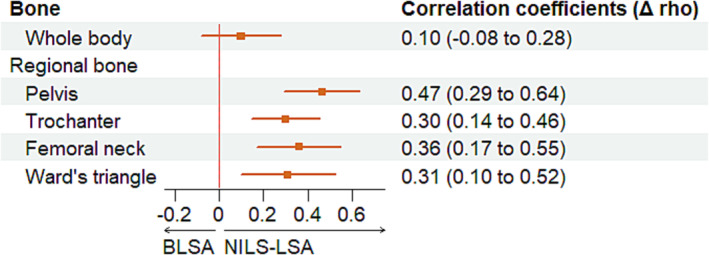
Cohort difference in the association between BMDs and appendicular lean mass in overall participants. Filled circles and bars indicate Δrho (rho from the NILS‐LSA study minus rho from the BLSA) and 95% confidential interval.

In the BLSA study, the rate of change in ALM was positively associated with a change in whole‐body BMD (rho = 0.30, *P* < 0.0001), pelvic BMD (rho = 0.24, *P* = 0.001), and trochanter (rho = 0.31, *P* < 0.0001; *Figure* *2*). In the NILS‐LSA study, the rate of change in ALM was positively associated with change of BMD in all bone sites (whole‐body, rho = 0.20, *P* = 0.001; pelvic, rho = 0.71, *P* < 0.0001; trochanter, rho = 0.61, *P* < 0.0001; femoral neck, rho = 0.46, *P* < 0.0001; Ward's triangle, rho = 0.45, *P* < 0.0001). Significant positive associations indicated that steeper declines in ALM were associated with proportionally steeper declines in BMD. The correlation coefficients in the NILS‐LSA study were larger than those of the BLSA in all bone regions except whole‐body BMD (*Figure* *3*).

### Sex‐specific longitudinal relationships between bone mineral density (BMD) and appendicular lean mass (ALM)

**Figure 4:**
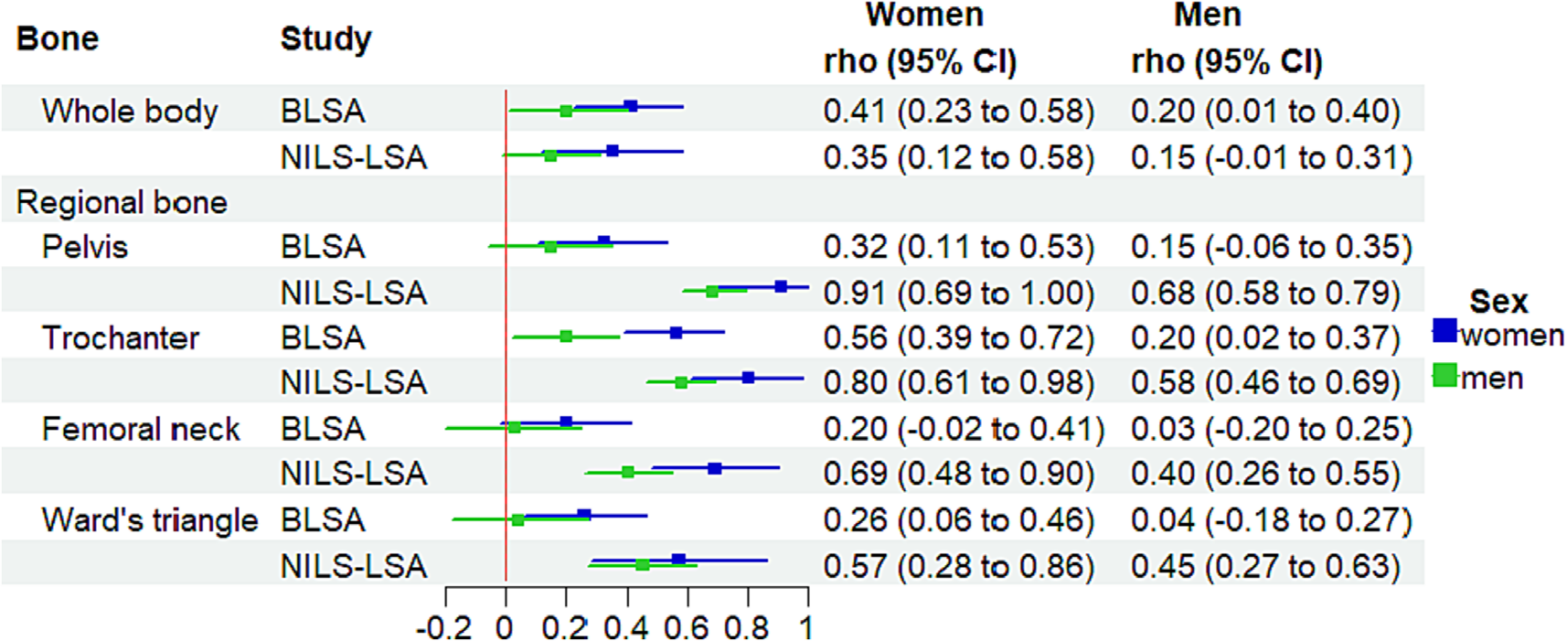
Results from cohort‐ and sex‐specific bivariate linear mixed‐effect model for the association between BMDs and appendicular lean mass in the BLSA and NILS‐LSA studies. Models were adjusted for age centred at 70 years, age squared, education year, weight, height, fat mass, smoking status, follow‐up time, and age*follow‐up time. In the BLSA study, models were further adjusted for race (black or not). Filled circles and bars indicate rho and 95% confidential interval.

**Figure 5:**
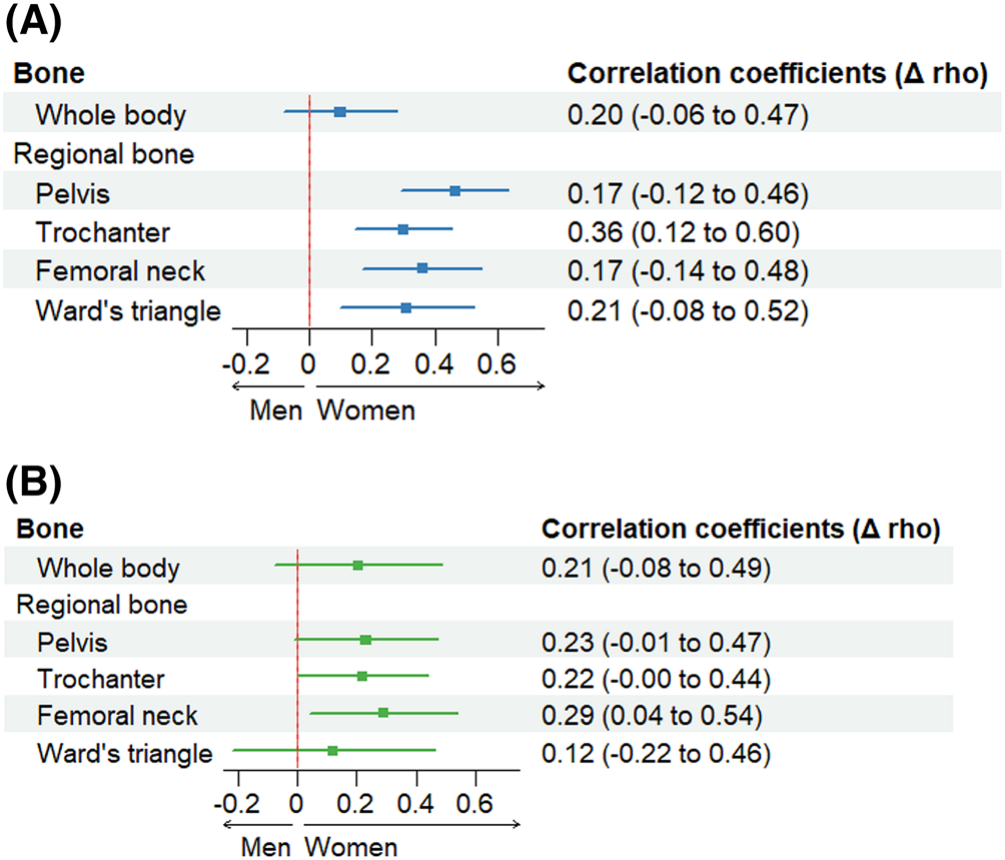
Sex differences in the association between BMDs and appendicular lean mass in the BLSA and NILS‐LSA studies. Filled circles and bars indicate Δrho (rho from women minus rho from men) and 95% confidential interval. (A) Results from the BLSA. (B) Results from the NILS‐LSA study.

In the BLSA, in women, the rate of change in ALM was positively associated with BMD change at all bone sites except the femoral neck (whole‐body BMD, rho = 0.41, *P* < 0.0001; pelvic BMD, rho = 0.32, *P* = 0.003; trochanter, rho = 0.56, *P* < 0.0001; Ward's triangle, rho = 0.26, *P* = 0.010; *Figure* *4*). In men, the rate of change in ALM was only positively associated with changes in whole‐body BMD and trochanter (whole body, rho = 0.20, *P* = 0.041; trochanter, rho = 0.20, *P* = 0.026). While in trochanter, the correlation coefficient was larger in women than in men (Δrho = 0.36, *P* = 0.003), there were no sex differences in other bone regions (*P* > 0.05; *Figure* *5A*).

In the NILS‐LSA study, in women, the rate of change in ALM was positively associated with a change in BMD at all bone sites (whole‐body BMD, rho = 0.35, *P* = 0.003; pelvic BMD, rho = 0.91, *P* < 0.0001; trochanter BMD, rho = 0.80, *P* < 0.0001; femoral neck BMD, rho = 0.69, *P* < 0.0001; Ward's triangle BMD, rho = 0.57, *P* = 0.0001) (*Figure* *4*). In men, the rate of change in ALM was positively associated with a change in pelvic BMD (rho = 0.71, *P* < 0.0001), trochanter BMD (rho = 0.61, *P* < 0.0001), and Ward's triangle BMD (rho = 0.45, *P* < 0.0001) (*Figure* *5B*). While in the femoral neck, the correlation coefficient was larger in women than in men (Δrho = 0.29, *P* = 0.02), there were no sex differences in other bone regions (*P* > 0.05).

### Longitudinal associations between bone mineral density and knee extension strength in overall samples

In the BLSA, no significant longitudinal associations were found between change in BMD and change in knee extension strength except trochanter (rho = 0.23, *P* = 0.028; *Table* *S2*). In the NILS‐LSA study, the rate of change in knee extension strength was positively associated with a change in BMD at all bone sites except whole body BMD (pelvic BMD, rho = 0.54, *P* < 0.0001; trochanter BMD, rho = 0.49, *P* < 0.0001; femoral neck BMD, rho = 0.30, *P* = 0.001; Ward's triangle BMD, rho = 0.29, *P* = 0.011).

### Sex‐specific longitudinal relationships between bone mineral density and knee extension strength

In the BLSA, similar to results from overall samples, no significant longitudinal associations were found except the association between the rate of change of BMD in trochanter and change in knee extension strength in women (rho = 0.35, *P* = 0.030; *Table* *S3*). In the NILS‐LSA study, in women, positive longitudinal associations between the rate of change in muscle strength and the rate of change of BMD in all bone sites except whole‐body BMD (rho ranging from 0.45 to 0.56, *P* < 0.01). In men, the rate of change in muscle strength was positively correlated with the rate of change in pelvic and trochanter BMDs (pelvis, rho = 0.59, *P* < 0.0001; trochanter, rho = 0.46, *P* < 0.0001).

### Longitudinal association between appendicular lean mass and muscle strength

In the BLSA study, the rate of change in ALM was correlated with the rate of change in muscle strength in overall (overall, rho = 0.27 [0.06–0.49], *P* = 0.014). In the NILS‐LSA study, the rate of change in ALM was correlated with the rate of change in knee extension muscle strength (overall, rho = 0.82 [0.63–1.00], *P* < 0.0001).

### Associations between IGF‐1 and the rate of change in appendicular lean mass or bone mineral density

Among the present analytic samples in the BLSA study, 657 participants had IGF‐1 data at least one visit (person‐visit, 1296). In the linear mixed‐effect model, an interaction term of time and sex was significant (*P* = 0.0008). In the sex‐specific linear mixed‐effect model, IGF‐1 decline was steeper in men than in women (β = −3.14, SE = 0.57, *P* < 0.0001; women, β = −0.42, SE = 0.55, *P* = 0.44). However, no significant association was observed between IGF‐1 and the rate of change in ALM or BMD in either sex (*P* > 0.05).

## Discussion

Using two cohorts from the United States and Japan, we examined longitudinal muscle mass–BMD association in community‐dwelling women and men aged 65 years or older. Larger loss in muscle mass co‐occurred with larger loss in BMD in most bone sites in both the BLSA and NILS‐LSA studies. However, the correlation coefficients in regional bones in the NILS‐LSA study were higher than those in the BLSA, implying population differences in the association between a loss of bone and loss of strength. Additionally, in both cohorts, the strength of the associations observed in women tended to be higher than in men, implying the association may differ by sex. We found a similar trend in the longitudinal association between muscle strength and BMD, but generally smaller correlation coefficients in both studies and all bone sites.

Interestingly, muscle mass loss co‐occurred with bone loss in two different cohorts, but the association was stronger in the NILS‐LSA study than in the BLSA. The correlation coefficients between bone and ALM tends to be lower in NILS‐LSA women, NILS‐LSA men, BLSA women and BLSA men, and this order is consistent with the order of lower BMD. This result suggests that even if individuals with higher ALM lose their muscle mass with age, this does not lead to weakening of the bones, resulting in a weaker correlation between BMD and ALM. Racial/ethnic differences in body composition are well known. Given the same BMI, Asians have higher percent fat than whites, and blacks have larger muscle mass and less fat mass than whites.^23^, ^24^ While body weight and fat mass strongly contribute to racial differences in BMD, large body size and composition differences exist between the two cohorts and may account for the detected differences.^25^ A recent epidemiological study reported racial differences in the association between sarcopenia and BMD; with blacks showing the strongest association followed by whites and Asians.^26^ Genomic studies implicate genetic and epigenetic heterogeneity as one explanation for these racial/ethnic differences.^27^, ^28^ For example, a GWAS (genome‐wide association studies) study indicated that the gene‐coded myostatin (*GDF8* gene, growth differentiation factor 8) is a shared mechanism in the pathogenesis of osteoporosis and sarcopenia. Myostatin is a suppressor of skeletal muscle formation but also acts as a bone loss factor in that it promotes osteoclastogenesis and inhibits and suppresses osteoblast differentiation.^29^, ^30^, ^31^ Environmental differences between the countries such as dietary habits, nutritional status, physical activity, and sunlight exposure may affect the cross‐cohort difference in the muscle–bone association.^32^ Future studies of the epigenetics of the muscle–bone association are needed to elucidate such cross‐country differences.

Longitudinal associations between ALM and BMD were consistent across the two cohorts, but not in the association between muscle strength and BMD. Skeletal muscle and bone tissues are thought to have a cross‐talk that allows them to respond harmonically to environmental stress and changes in body shape and function that occur with aging. However, major determinants for force production include muscle volume and neural factors. Our results showing that the association between ALM and muscle strength tended to be stronger in the NILS‐LSA study than the BLSA suggest that neural factors may differ by race/ethnicity. Our previous longitudinal study showed that muscle strength loss co‐occurs with regional brain atrophy in the frontal and temporal grey matter, superior and inferior frontal gyrus, and supramarginal gyrus.^33^ In these brain regions, the structure and function, and also age‐related changes are reported to differ between Asians and Caucasians.^34^, ^35^ One particularly interesting study reported that *APOE* ε4 carrier status is independently associated with age‐related brain volume change.^34^ Given that a combination of race/ethnicity and genetic factors can affect brain aging differently, the association between muscle strength and BMD in the BLSA may be more attenuated by racial diversity than in the NILS‐LSA study.

An important finding from the present study is that contrary to results from cross‐sectional studies, the bone–muscle association tended to be stronger in women than in men. The major differences between the present study and most previous work are the longitudinal nature of our study. Longitudinal studies are thought to provide better estimates of the true aging process because within‐subject changes are estimated, and each subject serves as their control. In contrast, cross‐sectional studies estimate the between subjects differences (not within‐subject changes), and both known and unknown confounders can bias the results. Although our results were consistent in that the bone–muscle association tended to be stronger in women than in men, the bivariate linear mixed‐effect model treats longitudinal changes as latent variables that may cause uncertainties with wider confidence intervals, resulting in no statistical significance in most bone regions in both cohorts. However, the present results provide new insight into sex differences in the bone–muscle association.

Multifaceted factors are implicated in sex‐specific bone–muscle association. In the aging process, along with age‐related depletion of sex hormones such as testosterone and oestrogen, men experience larger losses in muscle mass and strength with aging than women while women lose their bone mass and strength, especially after menopause.^36^, ^37^ Mechanical loading on bone tissues induces osteogenic response.^38^ However, in women, oestrogen deficiency after menopause reduces the activation level of oestrogen receptor (ER)‐α and its number, resulting in lower mechanosensitivity.^38^

Additionally, insulin‐like growth factor‐1 (IGF‐1) is implicated in controlling cell proliferation and differentiation of bone and muscle tissues.^39^ To date, no longitudinal studies have examined the association between IGF‐1 and bone or muscle mass. Using data from 1026 Japanese women and men aged 85–89 years, a recent cross‐sectional study indicated a negative association between serum IGF‐1 level and risk of osteoporosis and sarcopenia.^22^ Unfortunately, sex differences were not examined and sex was simply adjusted for as a confounder. Whether the role of IGF‐1 regulation in controlling cell proliferation and differentiation is equivalent in different cells has not been fully elucidated. Since IGF‐1 is regulated by testosterone whose circulation level sharply declines in men, we speculated that the effects of IGF‐1 depletion on bone and muscle losses may differ by sex. Indeed, using available data from the BLSA, we examined whether IGF‐1 is associated with the rate of change in ALM and BMD. However, contrary to our expectation, we did not find any significant associations possibly due to a relatively smaller sample size and shorter follow‐up time. Further studies are needed to identify the underlying mechanisms of intrinsic sex differences with a hypothesis‐free approach using multi‐omics data, which could inform how interventions for osteosarcopenia prevention should be tailored consistent with the specific underlying mechanisms in men and women.

An interesting finding from the present study is bone site specificity. Prior studies support the idea that the bone–muscle association is bone site‐specific. Among four regional bone sites examined (pelvis, trochanter, femoral neck, and Ward's triangle), the pelvis and trochanter were consistently the top two bone sites with the strongest associations with the rate of change in ALM and muscle strength in both cohorts, and sexes. Trochanter, femoral neck, and Ward's triangle are subregions of the hip. Correlation coefficient variations among the hip subregions may reflect the mechanical stimuli from closely aligned muscles including the gluteus medius, gluteus minimus, piriformis, obturator externus, and obturator internus. We cannot exclude the possibility of measurement error variability in the hip subregions may cause the difference as excess fat mass may cause measurement error of spine and hip BMD.^40^ This might be another possibility of smaller correlation coefficients in the BLSA that included a higher proportion of obese participants than in the NILS‐LSA study.

Our study has important strengths. The present analysis used data from two cohorts that have a similar women‐to‐men ratio, age ranges, the era of data collection, and follow‐up periods, but different characteristics in race, education level, and body size and composition. In the longitudinal association between the rate of change in muscle mass and BMD, while the correlation coefficients were higher in the NILS‐LSA study than those of the BLSA study, we found similar trends in terms of bone sites and sex differences, indicating that the results from one cohort study were replicated in the other cohort with different race. The availability of longitudinal data allowed the evaluation of change over time in both muscle mass and BMD. Our study also has limitations. Although our model was adjusted for possible covariates, the level of physical activity, dietary intake, and vitamin D status were not adjusted for because of the considerable number of missing data. The participants included in these analyses were healthy throughout the follow‐up visits. Thus, generalization of our findings to sicker and more disabled individuals is not possible.

## Conclusions

We found that muscle loss co‐occurred with bone loss in both cohorts, but the association in the NILS‐LSA was likely stronger than in the BLSA study, implying that the association may differ by country. Further longitudinal studies including a wide variety of races, lifestyles, and younger populations are needed to test the hypothesis that the bone–muscle association differs by countries and to identify country‐specific factors that may explain the detected difference may be considered as specific targets for preventive intervention aimed at preventing or slowing down osteosarcopenia.

## Funding

This research was supported by the Intramural Research Program of the National Institutes of Health, National Institute on Aging, JSPS KAKENHI (grant number 21K10505) and Keio University Global Research Institute Pre‐Start‐up Grant (FGCL000024). Research Funding for Longevity Sciences from the National Center for Geriatrics and Gerontology, Japan (grant number 21‐18).

## Conflict of interest

None declared.

## Supporting information


**Table S1.** Number of visits in the BLSA study and the NILS‐LSA study participants.
**Table S2.** The rate of change in muscle mass, muscle strength, and bone mineral density.
**Figure S1.** Distribution of follow‐up time (years) in the BLSA and the NILS‐LSA studies.
**Figure S2.** Results from cohort‐specific bivariate linear mixed‐effect model for the association between BMDs and knee extension muscle strength in the BLSA and NILS‐LSA studies.
**Figure S3.** Cohort‐ and sex‐specific results from bivariate linear mixed‐effect model for the association between bone mineral densities and muscle strength in the BLSA and NILS‐LSA studies.
