# Supplementary material for: Longitudinal association between muscle and bone loss: Results of US and Japanese cohort studies
Source: J Cachexia Sarcopenia Muscle. 2024 Feb 8;15(2):746–55. doi: 10.1002/jcsm.13438 (PMC10995282; doi:10.1002/jcsm.13438)
Supplement: Supplementary file 1 — Table S1. Number of visits in the BLSA study and the NILS‐LSA study participants. Table S2. The rate of change in muscle mass, muscle strength, and bone mineral density. Figure S1. Distribution of follow‐up time (years) in the BLSA and the NILS‐LSA studies. Figure S2. Results from cohort‐specific bivariate linear mixed‐effect model for the association between BMDs and knee extension muscle strength in the BLSA and NILS‐LSA studies. Figure S3. Cohort‐ and sex‐specific results from bivariate linear mixed‐effect model for the association between bone mineral densities and muscle strength in the BLSA and NILS‐LSA studies. [file JCSM-15-746-s001.docx]

**Supplementary materials**

| **Table S1. Number of visits in the BLSA study and the NILS-LSA study participants** | | | | | | | | | | | | | | | | | | |
| --- | --- | --- | --- | --- | --- | --- | --- | --- | --- | --- | --- | --- | --- | --- | --- | --- | --- | --- |
| **BLSA study** | | | | | | | | | | | | | | | | | | |
|  |  | Number of visits | | | | | | | | | | | | | | |  | |
|  |  | 1 | 2 | 3 | 4 | 5 | 6 | 7 | 8 | 9 | 10 | 11 | 12 | 13 | 14 | Total | |  |
| Women | n | 108 | 107 | 106 | 66 | 59 | 40 | 26 | 19 | 14 | 5 | 4 | 5 | 1 | 2 | 562 | |  |
|  | % | 19.22 | 19.04 | 18.86 | 11.74 | 10.50 | 7.12 | 4.63 | 3.38 | 2.49 | 0.89 | 0.71 | 0.89 | 0.18 | 0.36 | 49.78 | |  |
| Men | n | 125 | 80 | 92 | 74 | 60 | 56 | 36 | 15 | 11 | 9 | 3 | 4 | 2 | 0 | 567 | |  |
|  | % | 22.05 | 14.11 | 16.23 | 13.05 | 10.58 | 9.88 | 6.35 | 2.65 | 1.94 | 1.59 | 0.53 | 0.71 | 0.35 | 0.00 | 50.22 | |  |
| Total | n | 233 | 187 | 198 | 140 | 119 | 96 | 62 | 34 | 25 | 14 | 7 | 9 | 3 | 2 | 1129 | |  |
|  | % | 20.64 | 16.56 | 17.54 | 12.40 | 10.54 | 8.50 | 5.49 | 3.01 | 2.21 | 1.24 | 0.62 | 0.80 | 0.27 | 0.18 | 100 | |  |
|  |  |  |  |  |  |  |  |  |  |  |  |  |  |  |  |  | |  |
| **NILS-LSA study** | | | | | | | | | | | | | | | | | | |
|  |  | Number of visits | | | | | | | | | | | | | | |  | |
|  |  | 1 | 2 | 3 | 4 | 5 | 6 | 7 |  |  |  |  |  |  |  | Total | |  |
| Women | n | 295 | 227 | 150 | 107 | 105 | 82 | 61 |  |  |  |  |  |  |  | 1027 | |  |
|  | % | 28.72 | 22.10 | 14.61 | 10.42 | 10.22 | 7.98 | 5.94 |  |  |  |  |  |  |  | 49.78 | |  |
| Men | n | 227 | 186 | 182 | 118 | 103 | 81 | 74 |  |  |  |  |  |  |  | 971 | |  |
|  | % | 23.38 | 19.16 | 18.74 | 12.15 | 10.61 | 8.34 | 7.62 |  |  |  |  |  |  |  | 50.22 | |  |
| Total | n | 522 | 413 | 332 | 225 | 208 | 163 | 135 |  |  |  |  |  |  |  | 1998 | |  |
|  | % | 26.13 | 20.67 | 16.62 | 11.26 | 10.41 | 8.16 | 6.76 |  |  |  |  |  |  |  | 100 | |  |

| **Table S2. The rate of change in muscle mass, muscle strength, and bone mineral density** | | | | | | | | | | | | |
| --- | --- | --- | --- | --- | --- | --- | --- | --- | --- | --- | --- | --- |
|  | BLSA | | | | | | NILS-LSA | | | | | |
| The rate of change | Overall | | Women | | Men | | Overall | | Women | | Men | |
| (unit/year) | β(SE) | p-value | β(SE) | p-value | β(SE) | p-value | β(SE) | p-value | β(SE) | p-value | β(SE) | p-value |
| Muscle |  |  |  |  |  |  |  |  |  |  |  |  |
| Appendicular lean mass (kg) | -0.096(0.008) | <.0001 | -0.008(0.009) | 0.045 | -0.178(0.011) | <.0001 | -0.76(0.004) | <.0001 | -0.057(0.004) | <.0001 | -0.094(0.006) | <.0001 |
| Muscle strength† | -2.39(0.12) | <.0001 | -2.17(0.13) | <.0001 | -2.6(0.20) | <.0001 | -0.17(0.02) | <.0001 | -0.05(0.03) | 0.10 | -0.29(0.04) | <.0001 |
| Bone mineral density (g/cm2) | |  |  |  |  |  |  |  |  |  |  |  |
| Whole body | -0.003(0.000) | <.0001 | -0.004(0.000) | <.0001 | -0.002(0.000) | <.0001 | -0.002(0.000) | <.0001 | -0.002(0.000) | <.0001 | -0.002(0.000) | <.0001 |
| Pelvis | -0.004(0.000) | <.0001 | -0.005(0.000) | <.0001 | -0.004(0.000) | <.0001 | -0.004(0.000) | <.0001 | -0.006(0.000) | <.0001 | -0.003(0.000) | <.0001 |
| Femoral neck | -0.005(0.000) | <.0001 | -0.006(0.000) | <.0001 | -0.005(0.000) | <.0001 | -0.005(0.000) | <.0001 | -0.006(0.000) | <.0001 | -0.005(0.000) | <.0001 |
| Trochanter | -0.005(0.000) | <.0001 | -0.005(0.000) | <.0001 | -0.004(0.000) | <.0001 | -0.003(0.000) | <.0001 | -0.003(0.000) | <.0001 | -0.002(0.000) | <.0001 |
| Ward's triangle | -0.004(0.000) | <.0001 | -0.006(0.000) | <.0001 | -0.003(0.001) | <.0001 | -0.004(0.000) | <.0001 | -0.004(0.000) | <.0001 | -0.004(0.000) | <.0001 |
| †BLSA, Nm; NILS-LSA, kg | |  |  |  |  |  |  |  |  |  |  |  |


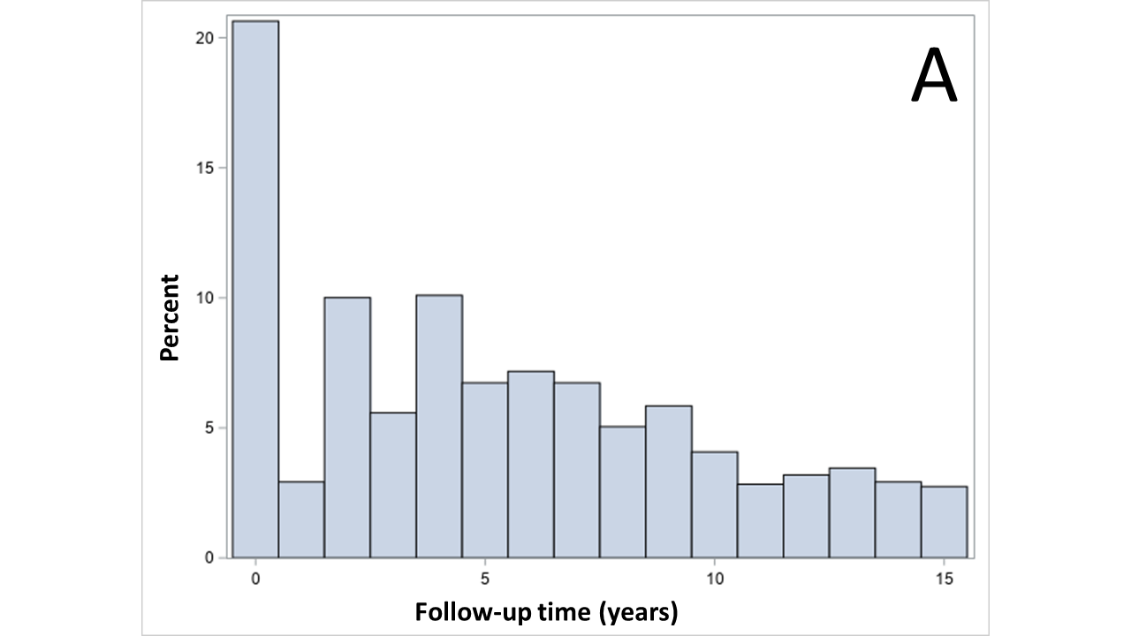

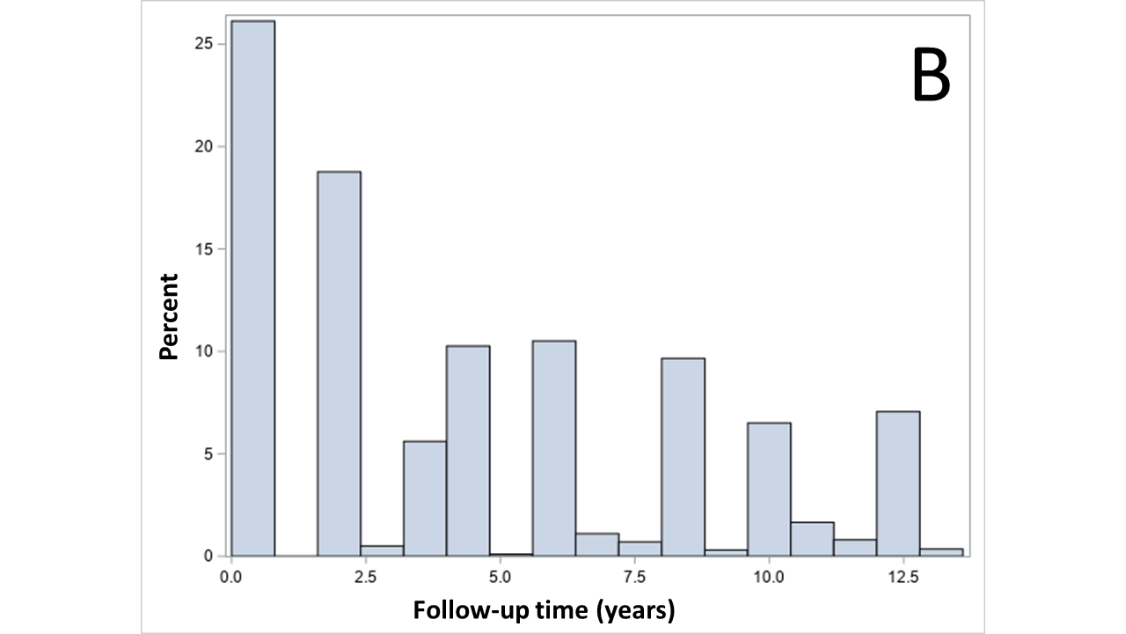


**Figure S1. Distribution of follow-up time (years) in the BLSA and the NILS-LSA studies**

A total of 1,129 participants were included from the BLSA study. Median follow-up time (years) was 4.60 (min-max, 0–15.35) years. A total of 1,998 participants were included from the NILS-LSA study. Median follow-up time (years) was 3.97 (min-max, 0–13.35) years.


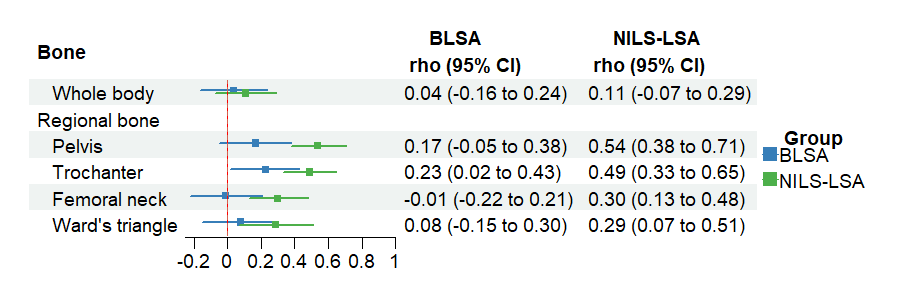


**Figure S2. Results from cohort-specific bivariate linear mixed-effect model for the association between BMDs and knee extension muscle strength in the BLSA and NILS-LSA studies**

**
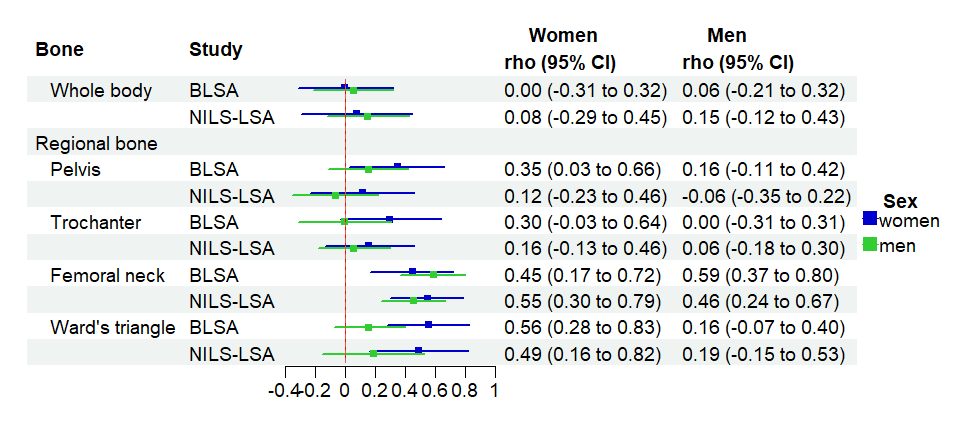
**

**Figure S3. Cohort- and sex-specific results from bivariate linear mixed-effect model for the association between bone mineral densities and muscle strength in the BLSA and NILS-LSA studies**
